# Internet-delivered cognitive behaviour therapy for chronic health conditions: self-guided versus team-guided

**DOI:** 10.1007/s10865-022-00346-x

**Published:** 2022-08-03

**Authors:** S. H. Mehta, M. Nugent, V. Peynenburg, D. Thiessen, G. La Posta, N. Titov, B. F. Dear, H. D. Hadjistavropoulos

**Affiliations:** 1 Department of Psychology, University of Regina, 3737 Wascana Parkway, Regina, SK S4S 0A2 Canada; 2 School of Psychological Sciences, Macquarie University, Sydney, NSW 2109 Australia

**Keywords:** Chronic conditions, Internet-delivered cognitive behaviour therapy, Anxiety, Depression

## Abstract

There is growing interest in offering Internet-delivered cognitive behaviour therapy (ICBT) to individuals with chronic health conditions, with this process often being guided by a single clinician. Due to lack of full time personnel, it is sometimes necessary to have multiple clinicians offer guidance or for no guidance to be offered. In this randomized trial, we compared team-guided ICBT (*n* = 90) to self-guided ICBT (*n* = 88). Participants completed measures at pre-, post-, and 3-months post-ICBT. Both groups showed similar rates of treatment completion and large improvements on depression and anxiety at post-treatment and follow-up. Unexpectedly, more participants in the self-guided versus team-guided condition showed clinically significant improvement on depression at post-treatment (76.5% vs 49.2%) and follow-up (70% vs 45.6%). Thus, team-guided ICBT may not provide significant benefits compared to self-guided ICBT. However, it may be an alternative approach to consider among a population of high risk individuals that wants or requires closer monitoring of symptoms.

*Trail registration* TRN: NCT03500237; Date: April 18, 2018.

## Introduction

Globally, chronic health conditions represent a significant problem requiring attention given their prevalence, costs, and association with disability (Vos et al., 2020). Of concern, as the number of physical health conditions increases, rates of comorbid mental health concerns increase, and the co-existence of mental and physical conditions is associated with reduced quality of life, increased suicidal ideation, and increased healthcare utilization (Dai et al., 2020). Although psychosocial interventions are increasingly incorporated into routine care of chronic health conditions, accessing support for comorbid mental health concerns remains challenging for individuals (Barnett et al., 2012), suggesting a need for innovative approaches. Barriers to receiving mental health care include costs and availability of services, time and location restraints, and concerns about stigma (Andersson & Titov, 2014). This is problematic, as addressing anxiety and depression outcomes among those with chronic health conditions has been shown to improve disability outcomes (Jasper et al., 2014; Vallejo et al., 2015), pain severity (Buhrman et al., 2004; Chiauzzi et al., 2010; Dear et al., 2013, 2015), and fatigue (Friesen et al., 2017; Williams et al., 2010).

The need for accessible treatment options has been further exacerbated by the 2020 pandemic, as individuals with chronic health conditions have been disproportionately burdened by COVID-19 morbidity and mortality (WHO, 2020; Camacho-Rivera et al., 2020). Face-to-face health service utilization has significantly decreased since the onset of the COVID-19 outbreak (Wong et al., 2020). Thus, those managing chronic health conditions have been encouraged to engage in telehealth appointments to minimize risk exposure while maintaining continuity of care (Horrell et al., 2021). Remote interventions can improve access to care for those who have been underserved, or those who are unable to attend in-person appointments.

Internet-delivered cognitive-behavioural therapy (ICBT) shows particular promise in improving psychological outcomes among individuals with chronic health conditions (Mehta et al., 2018). In ICBT, participants access treatment materials that have been arranged into lessons online, and either navigate the course materials on their own, or with guidance from a guide or clinician typically over several months (Anderson & Titov, 2014). Numerous meta-analyses have reported that guided ICBT results in larger clinical improvements in symptoms of depression and anxiety than self-guided ICBT (Andersson et al., 2019; Etzelmueller et al., 2020). A recent systematic review and meta-analysis showed that self-guided ICBT generally results in small effects for depression and moderate effects for anxiety among people with chronic health conditions; outcomes appear superior when participants receive guidance (Mehta et al., 2018). Studies among those with chronic health conditions also note the potential need for greater support i.e. patients requesting twice a week check in from their guide (Hadjistavropoulos et al., 2019; Mehta et al., 2018). The downside of offering clinician guidance is that it is associated with increased costs, and having the same designated clinician over several months also presents organizational challenges when there is staff turn-over, holiday and sick time. Furthermore, if clinicians work part-time, participants may have to wait for a response to questions until the clinician next works. Thus, in order to provide more intensive care (i.e., response to clients within one-business day for those with chronic conditions among clinical settings where full-time staff are not always available), it may require more than one clinician to offer support to participants completing ICBT, creating a team-guided approach. To our knowledge, team-guided ICBT, where therapeutic workload is spread across several clinicians depending on their availability (e.g., first available guide responds), has not been studied. The approach, however, has potential to improve outcomes over self-guided ICBT, while reducing or buffering against organizational challenges involved in having a single designated clinician work with clients throughout treatment.

In the present study, we were interested in comparing two methods of offering ICBT for people with chronic health conditions, namely ICBT that was team-guided versus ICBT that was self-guided. In the team-guided condition, varying clinicians (depending on work schedule) would send weekly emails to participants and also respond to any participant email received from the participant from the previous day (one-business-day response). Primary outcomes were depression and anxiety, and secondary outcomes were psychological distress, disability, quality of life, pain, fatigue, life satisfaction, self-efficacy, healthcare use, and mental health medication use. Outcomes were assessed at 8-weeks post-treatment and 3 months follow-up. Groups were also compared on objective engagement (e.g., lessons accessed) and treatment satisfaction. It was hypothesized that the team-guided approach would improve outcomes, engagement, and satisfaction above self-guided ICBT.

## Methods

### Ethics and trial registration

This study was approved by the University of Regina Ethics Board and was registered on clinicaltrials.gov: NCT03500237. Participants were recruited from across Canada via strategic advertising (posters, advertising cards, and presentations), media attention, and collaborations (e.g. course referrals) with service providers and non-governmental organizations representing and or providing services to people with chronic health conditions. In terms of sample size, using WebPower (Zhang & Yuan, 2018) with alpha = 0.05 and power = 0.80, we estimated that we required a total sample of 164 participants to detect a moderate between group effect size. We enrolled additional participants, as we anticipated that some patients would not begin the intervention after randomization, resulting in a total sample size of 195 participants.

### Intake and course progression

Participants first completed an online application on the Online Therapy Unit website (www.onlinetherapyuser.ca), a government- and research-funded unit that specializes in delivering ICBT for anxiety and depression routinely at no cost to participants. This online application collected background information and was followed by a telephone interview where screeners confirmed participants were: (1) at least 18 years of age; (2) residing in Canada; ((3) self-reported symptoms of anxiety/depression related to a chronic health condition, i.e.a physical health condition persisting for more than 3 months; (4) able to read and write in English; (5) consented to participate in ICBT; and (6) able to access the internet. During this interview, participants were excluded if they: (1) had a self-reported and/or clinician-identified difficulty with cognitive functioning that could impact their ability to fully engage in treatment (e.g., dementia); (2) were assessed as having a high risk of suicide; and (3) did not self-report having a chronic health condition. An emergency medical contact was required for participation in the course.

For the purposes of this study, chronic health conditions were characterized as any physical health condition sustained over 3 months or longer to distinguish conditions from those likely to be acute or transient (Perrin et al., 1993). See Table 1 for a list of common chronic health conditions. Participants who fully met eligibility criteria in the telephone screen were randomized into one of two groups (team-guided or self-guided) using Research Electronic Data Capture (REDCap) in a 1:1 ratio in blocks of 24 without matching. Telephone screeners did not know what condition the participant would be assigned until after the participant was accepted into the trial. Clinicians could not be blinded to randomization because the randomization group was related to the type of contact the participant received (i.e., team-guided vs self-guided).

**Table 1:** Patient characteristics at pre-treatment

	Combined (*n* = 178)		Team-Guided (*n* = 90)		Self-Guided (*n* = 88)	
	*n*	%	*n*	%	*n*	%
*Age*						
Mean (*SD*)	45.75 (13.61)	–	45.50 (12.98)	–	46.01 (14.30)	–
Range	19–78	–	20–76	–	19–78	–
*Sex*						
Female	136	76.4	70	77.8	66	75
Male	42	23.6	20	22.2	22	25
*Marital status*						
Married or common law	120	67.4	61	67.7	59	67.0
Single	38	21.3	18	20	20	22.7
Separated/divorced/widowed	20	11.2	11	12.2	9	10.2
*Education*						
High school diploma or less	31	17.4	13	14.4	18	20.5
Post high school certificate/diploma	46	25.8	23	25.6	23	26.1
Some university	32	18.0	18	20.0	14	15.9
University degree	69	38.8	36	40.0	33	37.5
*Employment status*						
Employed part-time/full time	74	41.6	37	41.1	37	42.0
Homemaker, student, retired	52	29.2	28	31.1	24	27.3
Unemployed	26	14.6	12	13.3	14	15.9
On disability	26	14.6	13	14.4	13	14.8
*Ethnicity*						
Caucasian	159	89.3	78	86.7	81	92.0
Indigenous/Metis/Inuit	8	4.5	7	7.8	1	1.1
Other	11	6.2	5	5.6	6	6.8
*Location*						
Large city (over 200,000)	81	45.5	42	46.7	39	44.3
Small/medium city	55	30.9	27	30.0	28	31.8
Town/village/farm	42	23.6	21	23.3	21	23.9
*Currently experiencing pain*	119	66.9	57	63.3	62	70.5
*Duration of chronic condition symptoms*^*a*^						
Less than 1 year	23	12.9	9	10.0	14	15.9
1–2 years	22	12.4	11	12.2	11	12.5
3–5 years	19	10.7	8	8.9	11	12.5
More than 5 years	74	41.6	40	44.4	34	38.6
Unknown duration	40	22.5	22	24.4	18	20.5
*Chronic health condition*						
Chronic pain^b^	139	28.6	71	27.2	68	30.2
High blood pressure	45	9.2	24	9.2	21	9.3
Diabetes	38	7.8	16	6.1	22	9.8
MI	36	7.4	15	5.7	21	9.3
Neurological conditions^c^	33	6.8	19	7.3	14	6.2
Asthma, COPD	27	5.6	14	5.4	13	5.8
Thyroid	20	4.1	14	5.4	6	2.7
Chronic skin disease	14	2.9	9	3.4	5	2.2
Other^d^	134	27.6	79	30.3	55	24.4
*Number of chronic conditions reported*						
One condition	42	23.6	19	21.1	23	26.1
Two conditions	39	21.9	17	18.9	22	25.0
Three conditions	39	21.9	25	27.8	14	15.9
Four or more conditions	58	32.6	29	32.2	29	33.0
*Mental health prescription medication use*	70	39.3	35	38.9	35	39.8
*Mental health characteristics*						
Infrequent use of some form of mental health treatment	62	34.8	30	33.3	32	36.4
Pre-treatment GAD-7 ≥ 10	99	55.6	54	60.0	45	51.1
Pre-treatment PHQ-9 ≥ 10	102	57.3	50	55.6	52	59.1
*Healthcare service use in the previous 3 months*						
GP/nurse	73	41.0	38	42.2	35	39.8
Psychologist/counselor/social worker	54	30.3	28	31.1	26	29.5
Psychiatrist	21	11.8	8	8.9	13	14.8
Medical specialist	14	7.9	6	6.7	8	9.1
*Referral source*						
Physician/medical health professional/medical clinic	65	36.5	36	40.0	29	33.0
Online source (e.g. website or email)	32	18.0	19	21.1	13	14.8
Friend/family/employer	29	16.3	12	13.3	17	19.3
Mental health professional/health region intake	24	13.5	10	11.1	14	15.9
Printed poster/card or media (e.g. newspaper, radio, TV, talks)	11	6.2	5	5.6	6	6.8
Other	17	9.6	8	8.9	9	10.3
*Mean treatment credibility (SD)*	21.25 (4.71)	–	21.60 (4.64)	–	20.90 (4.79)	–

GAD-7 = Generalized Anxiety Disorder-7; PHQ-9 = Patient Health Questionnaire-9

^a^Participants were asked an open-ended question regarding the duration of their chronic health condition. Responses were then coded based on these four durations. Responses that did not specify months or years were coded as ‘Unknown duration’

^b^Chronic pain included inflammatory arthritis, rheumatoid arthritis, fibromyalgia, neuropathic pain, musculoskeletal pain, migraine, POTS

^c^Neurological conditions included: stroke, spinal cord injury, multiple sclerosis, other neurological conditions

^d^Other included those conditions that had less than ten frequencies, such as cancer, immune disorder, gall bladder disorder; and those that self-selected other

Participants in the self-guided condition did not receive regular communication with a clinician and worked through the course materials independently. Each week, an assigned clinician, however, briefly reviewed the participant profile to monitor scores on symptom measures for signs the participant was experiencing increased distress (a 5-point increase in primary measure of depression or anxiety, or endorsed the presence of suicidal thoughts more than half the days per week on the primary depression measure). Participants who showed increased distress were contacted by the clinician by telephone to ensure safety. Otherwise, contact was only made if participants reported needing technical support to use the platform.

Those randomized to the team-guided condition received a once-weekly email from a team of up to five guides during treatment on the same day each week. Additionally, the team was instructed to check for messages from clients each day and send an additional email if they had received a message from the client. The number of guides that interacted with each client ranged from 2 to 5 with clients interacting with a mean of 3.79 guides during treatment (1.1% 2 guides, 32.2% 3 guides, 53.3% 4 guides, 13.3% 5 guides). Guides included 5 registered clinicians: 1 Master’s level registered social worker, 2 Master’s level certified counselors, 1 Master’s level registered psychologist, and 1 Master’s level provisionally registered psychologist. All guides had had at least 1 year experience delivering ICBT at the Online Therapy Unit. The guide with provisional status was supervised by a clinician with over 5 years of experience delivering ICBT. Training for all guides included a 2 days workshop on delivering the ICBT program, as well as supervised practice by a clinician with over 10 years’ experience in delivering ICBT. More information on the Online Therapy Unit is provided in Hadjistavropoulos et al. (2020a, 2020b). Guides also met monthly over the course of the study to discuss concerns in service delivery, ensure standardization of message delivery, and engage in strategies to help promote therapeutic alliance. Additionally, auditing of messages were conducted to ensure treatment fidelity. Guides primarily communicated with participants through emails on the treatment platform and were instructed to spend approximately 15 min per email to each participant on a weekly basis. In the email, guides highlighted content of the course, and offered feedback on symptom scores from the previous week. Guides were encouraged to build rapport with clients, answer client questions, assist clients in tailoring activities to their condition, reinforce completion of lessons, activities, and progress, and assist clients with challenges in applying skills and barriers to using skills. As noted above, guides sent one weekly message and then responded to any message the participant sent during the week within one-business day. Phone calls were rare and only made if risk was apparent as described above, if the participant had not logged in for over 7 days, or if the participant requested a phone call instead of email support. Guides were instructed to spend approximately 10 min on phone calls that were intended to facilitate understanding or provide psychoeducation. To appropriately test the team-guided approach, guides were scheduled each week to ensure participants had the experience of multiple guides, and the same guide did not send all emails or check in with the participant on the same day each week.

Both groups received access to a transdiagnostic ICBT program called the *Chronic Conditions Course*—developed by the eCentreClinic at Macquarie University, Australia and licensed by the Online Therapy Unit. The *Chronic Conditions Course* is made up of five easy to follow core lessons released sequentially during an 8-weeks period (Dear et al., 2018, 2022; Gandy et al., 2016; Mehta et al., 2020). Lessons are based on cognitive behaviour therapy and focus on helping participants learn concepts such as: identifying symptoms, thought challenging, de-arousal strategies, planning pleasant activities, graded exposure, activity pacing, and relapse prevention. Additional resources are made available to all participants in an open access format, which provide further detail on topics including sleep, assertiveness, attention, managing beliefs, managing chronic conditions, working with health professionals, mental skills, acute and chronic pain, panic and physical sensations, structured problem solving and worry time, and communication skills. Participants also have access to case stories designed to help participants learn to apply skills, and “do-it-yourself” guides that summarize lesson content and suggested homework for each lesson. All participants received weekly automated emails informing them of the content of the upcoming lesson.

### Measures

Primary and secondary measures were administered at pre-treatment, post-treatment, and 3-months-follow-up. Primary measures were also administered at the beginning of lessons 2–5 to facilitate symptom monitoring in both the self-guided and team-guided conditions.

#### Primary outcome measures

##### Patient health questionnaire-9 (PHQ-9)

The PHQ-9 is a nine-item self-report questionnaire designed to assess symptoms of depression over the past two weeks (Kroenke et al., 2001). Total scores range from 0 to 27, with scores ≥ 10 suggesting presence of major depressive disorder (Manea et al., 2012). The PHQ-9 has been shown to have high internal consistency (α = 0.86–0.89) and good construct validity (Kroenke et al., 2001).

##### Generalized anxiety disorder-7 (GAD-7)

The GAD-7 consists of 7 statements designed to measure symptoms of anxiety within the past two weeks. Total scores range from 0 to 21, with scores ≥ 10 indicating likely clinical levels of anxiety (Spitzer et al., 2006). Psychometric studies show that GAD-7 has excellent internal consistency (α = 0.92) and strong construct validity (Spitzer et al., 2006).

#### Secondary measures

##### World Health Organization disability assessment schedule (WHODAS)

The WHODAS is a 12-item self-report measure designed to assess several different aspects of functioning and disability over the last 30 days, including cognition, self-care, and life-activities (Axelsson et al., 2017). Total scores range from 0 to 48, with higher scores indicating increased disability (Axelsson, et al., 2017). Among those with physical health conditions, the WHODAS has been shown to have alpha estimates varying from 0.81 up to 0.96 (Saltychev et al., 2021).

##### The EuroQol-5D (EQ5D)

The EQ5D is a self-report measure used to assess quality of life (i.e., mobility, self-care, pain, and anxiety/depression) and overall health (rating scale of 0-*worst health* to 100-*best health*; EuroQol Research, 2015). The current study analyzed only data from the overall health scale (i.e., Visual Analogue Scale; VAS). The EQ-5D-5L demonstrates test–retest reliability, high internal consistency (α = 0.83), and good validity (Marti et al., 2016).

##### Kessler-10 distress scale (K10)

The K10 is a self-report measure designed to assess psychological distress over the past 30 days (Kessler et al., 2002). Total scores range from 0 to 40, with higher scores indicating more severe psychological distress. K10 presented excellent Cronbach’s alpha (α) (0.93).

##### Brief pain inventory (BPI)

The short-form BPI consists of four items related to pain severity and seven items related to pain interference (Cleeland & Ryan, 1994). A mean score is created for the pain severity (range: 0–10) and pain interference (range: 0–10) factors, with higher scores indicating more severe pain and greater pain-related interference, respectively. Of note, there was a problem with the administration of the BPI questions at 3-month follow-up, where only participants who answered “yes” to the first question were administered the rest of the BPI; because of this error it was not possible to compare pre- and post-BPI to the 3-months BPI. Good internal consistency, ranging from 0.80 to 0.87 are reported for pain severity items and from 0.89 to 0.92 for the interference items (Cleeland & Ryan, 1994).

##### Fatigue symptom inventory (FSI)

The FSI consists of 14 items designed to assess fatigue (Shahid et al., 2011). Consistent with the literature, responses for 13 of the items were summed to create a total fatigue score with a range from 0 to 130 (Shahid et al., 2011). Alpha coefficients for multi-item scales ranged from 0.84 to 0.96 (Donovan & Jacobsen, 2011).

##### Satisfaction with life scale (SWLS)

The SWLS consists of 5 items designed to assess global-life satisfaction (Deiner et al., 1985). Total scores on the SWLS range from 5 to 35, with higher scores indicating greater life satisfaction (Deiner et al., 1985). Internal consistency, estimated by coefficient α, ranged from 0.81 to 0.96 for the individual subscales (Deiner et al., 1985).

##### Self-efficacy for managing chronic disease-6 (SES-6)

This 6-item measure assesses participants’ confidence with managing chronic health conditions and their symptoms (Lorig et al., 2001). Responses to each question are made on a 10-point Likert scale; the measure is scored by calculating the average of the six items. Mean scores range from 0 to 10, with higher scores indicating greater confidence at managing chronic health conditions (Lorig et al., 2001). Cronbach's alpha of 0.88 was seen, minimal floor and ceiling effects were observed, and the measure was sensitive to change (Ritter & Lorig, 2014).

##### Treatment inventory of costs in psychiatric patients (TIC-P)

A modified version of the TIC-P (Bouwmans et al., 2013) was administered at pre-treatment and at 3-months follow-up. At pre-treatment, participants answered questions related to lifetime use and use in the past 3 months of mental health services (i.e., family doctor/walk-in clinic, psychiatrist, psychologist, social worker, counsellor, nurse/community nurse/psychiatric nurse, occupational therapist, medical specialist, or other health care professional), treatment programs (i.e., psychiatry day/part-time treatment program, alcohol or drug treatment program, self-help group, occupational stress injury program), hospital and crisis services (i.e., emergency room, ambulance, crisis service, and hospital admission), and mental health medication use (Bouwmans et al., 2013). At 3-months follow-up, they were asked if they had utilized any of the above services and mental health medication use in the previous 3-months period.

##### Credibility and expectancy questionnaire (CEQ)

The CEQ (Devilly & Borkovec, 2000) assesses participants’ perceived credibility and expected success of treatment and was administered at pre-treatment. The three items of the CEQ assessing treatment credibility were summed to create a total score ranging from 3 to 27, with higher scores indicating greater perceived credibility.

##### Treatment satisfaction

Participants completed the ICBT treatment satisfaction questions (Dear et al., 2011) at post-treatment. Items included forced-choice questions related to satisfaction with the course (i.e., overall, lessons/DIY guides, treatment platform) rated on a scale of 1 (*Very Dissatisfied*) to 5 (*Very Satisfied*), and yes/no questions about whether they would recommend the course to a friend, if the course was worth their time, and whether they experienced negative effects during the course.

##### Treatment engagement

Treatment engagement was assessed by the number of lessons accessed, days logging into the website, emails sent to guide, emails from guide to participant, and phone calls between participant and guide.

##### Guide feedback

At the end of the trial, all guides were asked to submit written feedback on the benefits and challenges of both team- and self-guided approaches. Feedback was summarized and reported in the results.

### Analyses

All analyses were completed using Statistical Package for the Social Sciences version 26 (SPSS) and R version 4.0.5. Internal consistency was examined on all measures and found acceptable (alpha > 0.83) at all time points. Demographic variables, such as age and injury type, were examined using descriptive statistics to describe the sample. Generalized estimating equations (GEE) (Hardin et al., 2013) were used to examine the effect of the *Chronic Conditions Course* on primary and secondary outcome measures. GEE analyses allow for measurements of changes over time while taking within-subject variance into consideration. GEE models for the VAS, SWLS, and SES-6 used a Gaussian distribution with identity link function. All other GEE models used a Gamma distribution with log link. All GEE models used an exchangeable working correlation structure and fully iterated jackknife estimates of variance. A number of statistical tests were employed based on GEE analyses to help interpret the results: (1) average percentage change across time with 95% confidence intervals; and (2) bias-corrected Cohen’s *d*_unb_ effect sizes for within and between group effects based on the estimated marginal means taken from the GEE models. Further analysis examined any potential differences between the team-guided condition and the self-guided condition, as well as clinically significant improvements of at least 30% or greater. As was previously recommended, outcome deteriorations greater than 30% for GAD-7 and PHQ9 outcomes were noted for post-treatment measures (Rozental et al., 2014). One-way ANOVAs were used to compare groups on continuous variables (e.g., number of messages sent to guide) and chi-square tests were used to compare groups on categorical variables (e.g., treatment satisfaction).

#### Missing data

39% (69/178) of participants were missing at least one primary measure at post-treatment and 26% (46/178) were missing at least one primary measure at follow-up. Multiple imputation were used to replace missing values before fitting the GEE models or calculating clinically significant improvements. In creating the imputation models, we found that course completion was significantly associated with missingness. Participants who did not complete at least 4 lessons were significantly less likely to have post-treatment (7.1% vs 77.2%, *p* < 0.001) or follow-up measures (16.4% vs 83.8%, *p* < 0.001). We were not able to adjust for course completion at post-treatment because there were only 3 participants who did not complete the course but did complete post-treatment measures. Therefore, when imputing missing values, we adjusted for randomization group and any observed measures for that participant at post-treatment, and when imputing follow-up measures, we additionally adjusted for course completion.

## Results

### Demographic variables

**Fig. 1:**
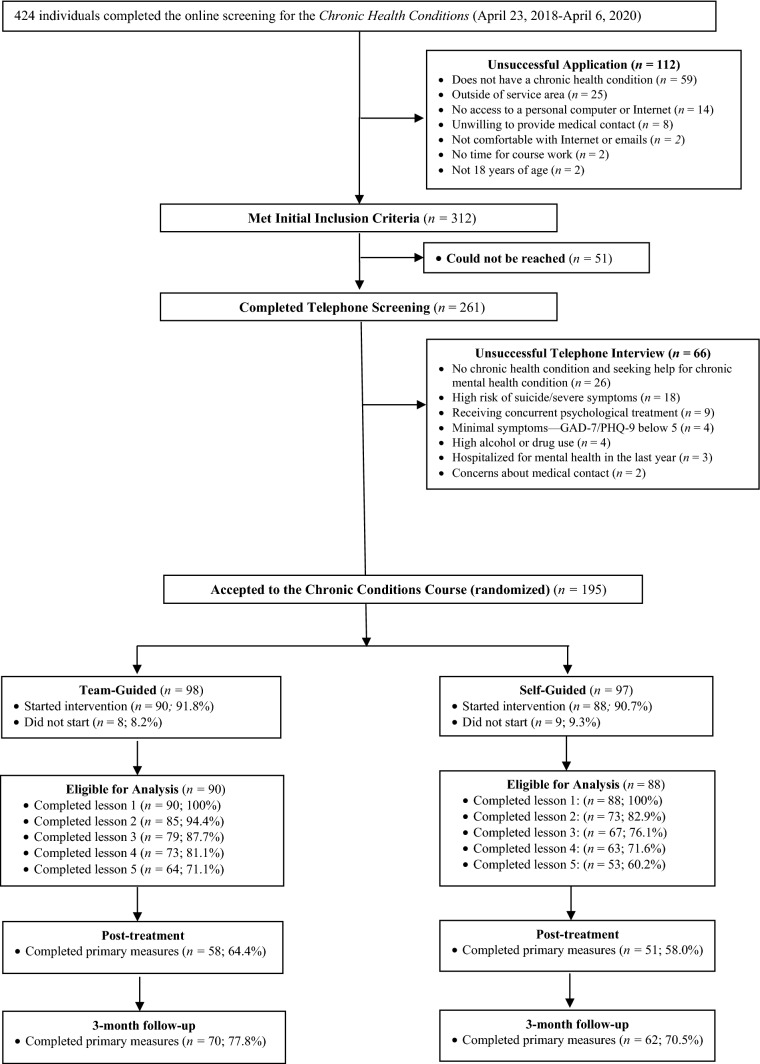
Patient flow from screening to 3 months follow-up

In total, 424 participants completed the online screen and were directed to the telephone screening. Of those participants, 261 completed the telephone screen and 195 participants were randomized into two groups. Of those randomized, 178 started the intervention, including 90 participants in the team-guided group, and 88 in the self-guided group. See Fig. 1 for participant flow. Table 1 includes the pre-treatment characteristics of the overall sample and each group. The average age of participants was 45.75 years (*SD* = 13.61), and most participants were female (76.4%, *n* = 136), married or common law (67.4%, *n* = 120), had more than a high school diploma (82.6%, *n* = 147), and Caucasian (89.3%, *n* = 159). Most participants also resided outside of large cities (55.5%, *n* = 97). At pre-treatment, 66.9% (*n* = 119) reported experiencing pain, 76.4% (*n* = 136) reported having more than one chronic health condition, and 41.6% (*n* = 74) reported having had a chronic health condition for more than 5 years. Depression was in the clinical range for 57.3% (*n* = 102) of participants while anxiety was in the clinical range for 55.6% (*n* = 99) of participants. No significant differences in baseline symptom scores were found between the randomization groups.

### Primary analyses

Results from the GEE models are given in Table 2. While statistically significant time effects were found on the PHQ-9 (*F*_(2, 319)_ = 60.1, *p* < 0.001) and GAD-7 (*F*_(2, 293)_ = 48.0, *p* < 0.001), no time*group interaction effects were found on the PHQ-9 (*F*_(2, 282)_ = 2.22, *p* = 0.11) or GAD-7 (F_(2, 284)_ = 1.23, *p* = 0.29). Cohen’s *d*_unb_ within-group effect sizes from pre-treatment to post-treatment and follow-up were medium to large in the team-guided condition, and large in the self-guided condition and overall sample.

**Table 2:** Estimated marginal means, 95% confidence intervals, percentage changes, and effect sizes (Cohen’s d) for the primary and secondary outcomes by group pooled imputations under MAR assumption

	Estimated marginal means			Percentage changes from pre-treatment		Within-group effect sizes from pre-treatment		Post-treatment between group effect size
	Pre-treatment	Post-treatment	3-months follow-up	To post-Treatment	To 3-months follow-up	To post-treatment	To 3-months follow-up	
Primary outcomes								
PHQ-9								
Combined	12.20 (5.47)	7.35 (4.85)	7.33 (4.59)	40 [33, 46]	40 [34,46]	0.94 [0.72, 1.16]	0.96 [0.74, 1.18]	N/A
Team	11.78 (5.53)	7.77 (4.97)	7.67 (4.56)	34 [24, 44]	35 [26, 44]	0.76 [0.46, 1.06]	0.81 [0.50, 1.11]	0.18 [− 0.12, 0.47]
Self	12.64 (5.40)	6.91 (4.70)	6.98 (4.62)	45 [37, 54]	45 [36, 53]	1.13 [0.81, 1.44]	1.12 [0.80, 1.44]	
GAD-7								
Combined	11.08 (5.06)	6.69 (4.77)	6.24 (4.44)	35 [24, 45]	43 [33, 52]	0.89 [0.67, 1.11]	1.02 [0.79, 1.24]	N/A
Team	10.99 (5.33)	7.19 (4.96)	6.30 (4.64)	45 [34, 55]	45 [35, 54]	0.73 [0.43, 1.04]	0.93 [0.63, 1.24]	0.22 [− 0.08, 0.51]
Self	11.18 (4.80)	6.16 (4.52)	6.17 (4.24)	15 [6, 24]	22 [13, 31]	1.07 [0.76, 1.39]	1.10 [0.78, 1.42]	
Secondary outcomes								
K10								
Combined	27.05 (7.08)	22.73 (7.59)	22.33 (7.13)	16 [11, 21]	17 [13, 22]	0.59 [0.38, 0.80]	0.66 [0.45, 0.88]	N/A
Team	27.08 (7.19)	24.02 (8.07)	22.75 (7.37)	11 [4, 18]	16 [9, 23]	0.40 [0.10, 0.69]	0.59 [0.29, 0.89]	0.35 [0.05, 0.64]
Self	27.02 (7.00)	21.41 (6.83)	21.90 (6.86)	21 [14, 27]	19 [13, 25]	0.81 [0.50, 1.12]	0.74 [0.43, 1.04]	
WHODAS								
Combined	16.53 (9.21)	14.04 (9.63)	12.91 (9.18)	15 [6, 24]	22 [13, 31]	0.26 [0.06, 0.47]	0.39 [0.18, 0.60]	N/A
Team	16.78 (9.61)	14.40 (10.20)	13.46 (9.72)	14 [1, 27]	20 [7, 32]	0.24 [− 0.05, 0.53]	0.34 [0.05, 0.64]	0.07 [− 0.22, 0.37]
Self	16.28 (8.84)	13.67 (9.05)	12.35 (8.61)	16 [3, 29]	24 [12, 36]	0.29 [− 0.01, 0.59]	0.45 [0.15, 0.75]	
EQ-5D VAS								
Combined	54.48 (21.72)	64.45 (20.10)	57.99 (20.93)	− 18 [− 25, − 12]	− 6 [− 13, 0]	− 0.48 [− 0.69, − 0.26]	− 0.16 [− 0.37, 0.04]	N/A
Team	53.54 (22.67)	62.32 (21.66)	55.45 (22.14)	− 16 [− 26, − 7]	− 4 [− 13, 6]	− 0.39 [− 0.69, − 0.10]	− 0.08 [− 0.38, 0.21]	− 0.21 [− 0.51, 0.08]
Self	55.44 (20.80)	66.63 (18.13)	60.58 (19.34)	− 20 [− 29, − 11]	− 9 [− 19, 0]	− 0.57 [− 0.87, − 0.27]	− 0.25 [− 0.55, 0.04]	
FSI								
Combined	66.54 (26.19)	59.15 (29.89)	58.04 (29.83)	11 [4, 18]	13 [5, 20]	0.26 [0.05, 0.47]	0.30 [0.09, 0.51]	N/A
Team	65.61 (26.77)	61.74 (30.00)	58.78 (29.44)	6 [− 4, 16]	10 [0, 21]	0.14 [− 0.16, 0.43]	0.24 [− 0.05, 0.54]	0.18 [− 0.12, 0.47]
Self	67.50 (25.71)	56.48 (29.67)	57.25 (30.31)	16 [6, 26]	15 [4, 27]	0.40 [0.10, 0.69]	0.36 [0.07, 0.66]	
SWLS								
Combined	17.02 (7.61)	18.55 (7.68)	18.89 (8.03)	− 9 [− 16, − 2]	− 11 [− 19, − 3]	− 0.20 [− 0.41, 0.01]	− 0.24 [− 0.45, − 0.03]	N/A
Team	16.81 (7.37)	18.16 (7.86)	18.70 (8.49)	− 8 [− 19, 2]	− 11 [− 23, 0]	− 0.18 [− 0.47, 0.12]	− 0.24 [− 0.53, 0.06]	− 0.10 [− 0.39, 0.19]
Self	17.23 (7.88)	18.94 (7.51)	19.09 (7.57)	− 10 [− 20, 0]	− 11 [− 21, 0]	− 0.22 [− 0.52, 0.07]	− 0.24 [− 0.54, 0.06]	
Self-efficacy								
Combined	4.80 (2.03)	5.95 (2.08)	5.55 (2.54)	− 24 [− 32, − 17]	− 16 [− 26, − 5]	− 0.56 [− 0.78, − 0.35]	− 0.33 [− 0.54, − 0.12]	N/A
Team	4.86 (2.03)	5.73 (2.13)	5.53 (2.47)	− 18 [− 28, − 8]	− 14 [− 26, − 2]	− 0.42 [− 0.71, − 0.12]	− 0.30 [− 0.59, 0.00]	− 0.22 [− 0.52, 0.07]
Self	4.73 (2.03)	6.19 (2.02)	5.57 (2.61)	− 31 [− 42, − 20]	− 18 [− 34, − 2]	− 0.72 [− 1.03, − 0.42]	− 0.36 [− 0.65, − 0.06]	
BPI severity^a^								
Combined	3.23 (2.36)	2.97 (2.25)	–	8 [− 3, 19]	–	0.11 [− 0.10, 0.32]	–	N/A
Team	3.11 (2.37)	3.02 (2.30)	–	3 [− 14, 19]	–	0.04 [− 0.25, 0.33]	–	0.04 [− 0.25, 0.34]
Self	3.35 (2.37)	2.93 (2.21)	–	13 [− 2, 27]	–	0.18 [− 0.11, 0.48]	–	
BPI interference^a^								
Combined	4.05 (2.78)	3.50 (2.93)	–	14 [2, 26]	–	0.19 [− 0.01, 0.40]	–	N/A
Team	3.91 (2.80)	3.53 (3.00)	–	10 [− 8, 27]	–	0.13 [− 0.16, 0.42]	–	0.02 [− 0.27, 0.32]
Self	4.20 (2.76)	3.46 (2.88)	–	18 [1, 34]	–	0.26 [− 0.04, 0.56]	–	

Team, Team-Guided; Self = Self-Guided; PHQ-9, Patient Health Questionnaire-9; GAD-7, Generalized Anxiety Disorder-7; K10, Kessler 10 Item Scale; WHODAS = World Health Organization Disability Assessment Schedule; EQ5D VAS = 5-level EuroQol 5D version Visual Analogue Scale; FSI = Fatigue Symptom Inventory; SWLS = Satisfaction with Life Scale; Self-Efficacy, Self-Efficacy for Managing Chronic Disease; BPI, Brief Pain Inventory

^a^There was a problem with the administration of the BPI questions at 3-months follow-up, where only patients who answered "yes" to the first question were administered the rest of the BPI, and as a result we can't compare the 3-months BPI to data collected at pre- and post-treatment

The self-guided condition had significantly higher rates of PHQ-9 clinically significant improvement at both post-treatment (76.5% vs 49.2%, *p* = 0.004) and follow-up (70.0% vs 45.6%, *p* = 0.005). Group differences in GAD-7 improvement rates (71.9% for self-guided and 63.4% for team-guided) and in PHQ-9 or GAD-7 deterioration rates (PHQ-9: 4.7% for self-guided and 6.7% for team-guided; GAD-7: 8.5% for self-guided and 6.9% for team-guided) were not significant.

On the secondary measures, the GEE analysis found statistically significant time effects on the WHODAS (*F*_(2, 234)_ = 15.5, *p* < 0.001), EQ-5D VAS (*F*_(2, 316)_ = 15.3, *p* < 0.001), K10 (*F*_(2, 253)_ = 28.9, *p* < 0.001), BPI Interference (*F*_(2, 132)_ = 8.0, *p* = 0.006), FSI (*F*_(2, 258)_ = 9.1, *p* < 0.001), SWLS (*F*_(2, 238)_ = 5.7, *p* = 0.004), and SES-6 (*F*_(2, 271)_ = 23.0, *p* < 0.001). Time effects were not significant on the BPI Severity (*F*_(2, 190)_ = 3.6, *p* = 0.06). There were no significant time*group interaction effects on any secondary measure (*p* range 0.05–0.90). *d*_unb_ within-group effect sizes were medium to large on the K10, medium to small on the SES-6, medium to very small on the EQ-5D VAS, and small to very small on all other secondary measures. There was a statistically significant between-group *d*_unb_ effect size at post-treatment on the K10, with the team-guided group having higher K10 post-treatment scores suggesting poorer outcomes (24.02 vs 21.41, *d*_unb_ = 0.35).

#### Health service and medication use

No differences were found from pre-treatment to 3-months follow-up in the proportion of participants who saw a family doctor/walk-in clinic/nurse/other health professional (43.8%, *n* = 78/178 vs 39.5%, *n* = 50/127, *p* = 0.75), or psychiatrist (11.8%, *n* = 21/178 vs 14.2%, *n* = 18/127, *p* = 1.00). However, there was an increase in the proportion of participants who reported seeing a psychologist/counsellor/social worker, from 30.3% (*n* = 54/178) to 39.4% (*n* = 50/127), *p* = 0.04. Health service usage did not differ between team-guided and self-guided participants at 3-months follow-up (*p* range: 0.19–0.89). Further, no differences were found from pre-treatment to 3 months follow-up in the proportion of participants who reported taking medication for their mental health in the previous 3 months (39.3%, *n* = 70/178 vs 37.0%, *n* = 47/127, *p* = 1.00). Medication use at 3-months follow-up did not differ between team-guided (35.8%, *n* = 24/67) and self-guided (38.3%, *n* = *p* = 0.77) conditions.

#### Program engagement

Table 3 displays program engagement measures separated by group, with some significant differences found. As would be expected, team-guided participants received more messages from the guide than the self-guided participants (an average of 10.99 vs 0.45), as well as more phone calls (1.61 vs 0.59). Participants sent more messages to guides in the team-guided condition than self-guided condition (3.27 vs 0.47) and logged in a greater number of times (17.79 vs 14.18). No differences were found in terms of completion rates for the first four lessons (*p* = 0.14) or all five lessons (*p* = 0.13) or for completion of the post-treatment (*p* = 0.38) or 3-months (*p* = 0.27) primary outcome measures.

**Table 3:** Program engagement and satisfaction

	Combined (*n* = 178)		Team-guided (*n* = 90)		Self-guided (*n* = 88)		Significance
	*n*	%	*n*	%	*n*	%	
Completion of 4 lessons	136	76.4	73	81.1	63	71.6	(1, N = 178) = 2.22, *p* = .14
Completion of 5 lessons	117	65.7	64	71.1	53	60.2	(1, N = 178) = 2.34, *p* = .13
Completion of post- treatment primary measures^a^	109	61.2	58	64.4	51	58.0	(1, N = 178) = .79, *p* = .38
Completion of 3-months primary measures^a^	132	74.2	70	77.8	62	70.5	(1, N = 178) = 1.24, *p* = .27
Mean number of log-ins (*SD*)	16.01 (9.75)	–	17.79 (9.59)	–	14.18 (9.64)	–	*F*_(1,177)_ = 6.27, *p* = .01
Mean number of phone calls with guide (*SD*)	1.11 (1.40)	–	1.61 (1.68)	–	0.59 (0.77)	–	*F*_(1,177)_ = 26.93, *p* < .001
Mean written messages sent to guide (*SD*)	1.88 (3.16)	–	3.27 (3.80)	–	0.47 (1.24)	–	*F*_(1,177)_ = 43.40, *p* < .001
Mean written messages received from guide (*SD*)	5.78 (5.74)	–	10.99 (3.03)	–	0.45 (0.92)	–	*F*_(1,177)_ = 977.74, *p* < .001

	Combined (*n* = 103)		Team-Guided (*n* = 57)		Self-Guided (*n* = 46)		Significance
	*n*	%	*n*	%	*n*	%	
# said course was worth their time	99	96.1	54	94.7	45	97.8	*F*_(1,102)_ = .64, *p* = .43
# said they would recommend a friend	98	95.1	53	93.0	45	97.8	*F*_(1,102)_ = 1.28, *p* = .26
# said ‘Satisfied’ or ‘Very Satisfied’ with course	90	87.4	49	86.0	41	89.1	*F*_(1,102)_ = .23, *p* = .64
# that experienced negative effects during course	20	19.4	9	15.8	11	23.9	(1, N = 103) = 1.07, *p* = .30

^a^Primary measures included the Patient Health Questionnaire-9 (PHQ-9) and General Anxiety Disorder-7 (GAD-7)

#### Program satisfaction and negative effects

Table 3 shows ratings of participants’ satisfaction and negative effects. Group differences were not significant (*p* range: 0.26–0.64). As demonstrated in Table 3, 96.1% (*n* = 99) of participants completing measures reported that the course was worth their time taking, 95.1% (*n* = 98) said that they would recommend the course to a friend, and 87.4% (*n* = 90) said that they were either “satisfied” or “very satisfied” with the course. Out of 103 participants who responded, 20 (19.4%) reported that they had experienced unwanted negative effects during the course, with most of those participants reporting that the negative effect had a “moderate” impact on their lives (65.0%, 13/20). The most common negative effect reported was a feeling of pressure or guilt related to the course’s expectations (40.0%, 8/20; e.g., “Slight stress with getting the lessons done”^ID 8205^). Other negative effects were related to an increase in symptoms (20.0%, 4/20), external stressors (15.0%, 3/20), health-related limitations (15.0%, 3/20), and difficulties with self-reflection (10.0%, 2/20).

#### Guide feedback

In terms of the team-guided approach, guides reported that because they had a shared caseload, it was easier to seek support from each other when they experienced challenges delivering support to participants (e.g., were uncertain how to respond to a unique question about a chronic health condition). In the self-guided approach, guides reported that monitoring of symptoms was typically brief and efficient, and the need to follow-up with participants was rare. In both approaches, the guides noted that having a shared caseload often resulted in guides feeling unfamiliar with the participants they were emailing. They noted that before emailing participants, they would review the participant files to better understand the participants’ treatment experience. This process was especially challenging within the team-guided approach, as guides felt the need to thoroughly review participant files during each contact to ensure they were familiar with previous interactions and progress with a different guide, and noted this process took additional time. Concerns about unfamiliarity were less common in the self-guided approach, given that contact would only happen in exceptional circumstances (i.e., increased suicide risk), but guides noted that they also required additional time to review the participant file if an elevated score was found.

## Discussion

The current study is the first to examine the efficacy of a team-guided approach compared to a self-guided approach when delivering ICBT for people with chronic health conditions. Improvements were observed across both groups for all primary outcome measures and most secondary outcomes, and no significant differences in outcomes were observed between the groups. The outcomes were large for depression and anxiety (our primary outcome measures); moderate for psychological distress, self-efficacy, and the EQ-5D VAS; small for fatigue, life satisfaction, and pain interference; and non-significant for pain at post-treatment. The findings were unexpected for two reasons. Firstly, a recent systematic review and meta-analysis showed that self-guided ICBT generally results in small effects for depression and moderate effects for anxiety (Mehta et al., 2018); as such, finding large effects for depression and anxiety in the current study was unexpected. A recent study examining the *Chronic Conditions Course* found small but significant improvements in depression (d = 0.47), anxiety (d = 0.32), and disability (d = 0.17) at post-treatment in the ICBT group compared to a waitlist control group (Dear et al., 2022). Secondly, the same systematic review suggested that ICBT outcomes are superior when participants receive guidance (Mehta et al., 2018), and as noted above, the team-guided approach was not superior to the self-guided approach. Also unexpected was the fact that the self-guided group had a greater portion of participants report a 30% decrease in depressive symptoms compared to the team-guided group at post-treatment (76.5% vs 49.2%) and at 3-months follow-up (70.0% vs 45.6%). Several factors may have contributed to the current findings.

One factor may be the relatively high level of adherence observed among both groups, with no differences in completion of lessons (overall 76.4% completed four lessons and 65.7% completed all five lessons). It was surprising that the team-guided approach did not result in significantly greater completion of lessons than the self-guided approach given that past studies find greater adherence to ICBT in guided programs compared to self-guided programs (Richards & Richardson, 2012). Previous systematic reviews also found that small difference in adherence amongst self- and guided-ICBT groups resulted in slight differences in effect size between the groups (Cuijpers et al. 2009). Previously, only one study evaluated the efficacy of guided ICBT compared to self-guided ICBT (Dear et al., 2015). The study found no significant difference between the two groups in outcomes. Similar high levels of adherence were seen in the study for both guided and self-guided ICBT, 82% vs 78% respectively (Dear et al., 2015).

Additionally, although self-guided participants worked primarily independently, they all received a telephone interview at pre-treatment, which can help prepare participants for treatment (Titov et al., 2015). In addition, they all received automated messages and had a clinician that monitored their progress and symptoms, which may have led them to feel more supported than if the program was strictly self-guided. Furthermore, the treatment content and methods for facilitating engagement have been carefully developed over numerous trials and consistent with this, we found that all participants were highly satisfied with the program materials. These factors have been previously recognised as important in facilitating improvements in self-guided interventions (Andersson & Titov, 2014; Dear et al., 2016). Recent research suggests that “alliance with the program” is predictive of outcomes in ICBT (Zalaznik et al., 2021) and thus perhaps it was high alliance with the program in both the self-directed and team-guided groups that accounts for lack of differences between groups.

It is possible that the team-guided approach resulted in a level of support that was not well-matched to the participants’ needs. Participants received a minimum of once-weekly contact, but could receive additional responses within one-business day if they messaged their guide. In previous research on once-weekly therapist support plus one-business-day responses to participant emails, no benefits were found for this type of contact compared to once-weekly support in a general population without chronic health conditions (Hadjistavropoulos et al., 2020a, 2020b). Furthermore, in one previous study, there was some indication that increased therapist contact was associated with participants feeling lower confidence in their ability to manage symptoms than once-weekly contact (Hadjistavropoulos et al., 2020a, 2020b). Therefore, it is possible that increasing contact beyond once weekly in the team-guided appropriate in this study inadvertently undermined the benefits of team-guided support.

The other factor that could be at play is that receiving feedback from several different clinicians could have altered the participant-clinician rapport. There is conflicting evidence about the role of therapeutic alliance on participant outcomes in internet interventions; there is at least some research to suggest that some aspects of alliance (e.g., agreement with tasks and goals) are related to outcomes of ICBT (see Berger et al., 2017). Although the guides in the current study attempted to use strategies to enhance alliance, it may be that inconsistent contact with team-guided clinicians altered perceptions of therapeutic alliance, thereby affecting participant outcomes. Clinicians in the current study did not specifically report on alliance with participants—their feedback indicated they experienced challenges following participant progress in the team-guided condition which may indicate some alterations to alliance. As no previous research has evaluated the role of therapeutic alliance when ICBT is delivered by multiple clinicians, future research on its role is warranted.

The study was not without its limitations. First, although completion of 3-months follow-up measures was quite high (team-guided 77.8%; self-guided 70.5%), post-treatment completion rates (64.4% team-guided; 58.0% self-guided) were lower than we anticipated given our previous research (Hadjistavropoulos et al., 2018). Completion rates may have been lower at post-treatment because participants had to log back onto the website to complete them, whereas at 3-months follow-up, they were emailed a link to complete them. As with all studies, there is always a question about generalizability of study findings to other settings and thus replication of findings is needed. We also did not measure therapeutic alliance with clinicians and this may have provided useful information that could assist with interpreting findings. We were also not powered to identify small differences between groups and only tracked mental health medication use at 3-months follow-up. Further, the study involved a 3-months follow-up and it is possible that differences may have emerged longer term. Finally, it has been noted that the use of brief screening measures (e.g., the PHQ-9 and GAD-7) may over-estimate the prevalence of depression and anxiety and have the potential to pathologize normal human distress (Titov & Andersson, 2021).

Despite these limitations, the current study has several strengths. This is the first trial evaluating the effects of a team-guided approach when delivering ICBT, and thus adds to the literature on models for offering ICBT. The study evaluated ICBT for diverse chronic health conditions, which is often not examined in the literature, with most previous research focussing on ICBT for single health conditions (e.g., pain, cancer; see Mehta et al., 2018). The study also evaluated several outcome measures, and showed that outcomes are particularly strong for depression, anxiety, psychological distress and self-efficacy, while improvements for disability, fatigue, pain-interference and life satisfaction are considerably smaller. Pain intensity itself was not impacted by the chronic conditions course and may represent an opportunity to improve the course given that 66.9% of participants endorsed having difficulties with pain.

Several directions for research related to the team-guided approach exist. As noted above, if the team-guided approach is used in the future, it would be valuable to study if there is a way to implement the approach that would improve outcomes beyond what was found in the current approach, such as consistently having two designated clinicians offer support with a predictable schedule known to patients rather than an unknown, unpredictable schedule and typically 3 to 4 clinicians providing support as was done in this study. It would also be valuable to examine if the team-guided approach may mitigate adverse events among those with greater levels of suicide risk or severe symptoms compared to the self-guided approach. Furthermore, with larger samples it would be valuable to explore if clinician support is more important for some chronic health conditions than others. It would also be valuable to explore if the self-guided condition would be effective if it did not involve the initial telephone interview or involve monitoring of participant symptoms during treatment. Future research could also examine the role of participant preferences, by exploring participant preference for the self-guided vs team-guided version. Additionally, research examining longer-term outcomes beyond 3-months follow-up is warranted.

In conclusion, the results from the current study suggest that both team-guided and self-guided ICBT for chronic health conditions have similar effects in improving symptoms of anxiety and depression. Although, the current study shows non-inferiority of self-guided vs team-guided approach, the team-guided approach may still be an alternative approach to consider among a population of high risk individuals that wants or requires closer monitoring of symptoms (*n* = 99) perhaps related to symptom severity of risk. The use of team-guided approaches may be a more preferred option than self-guided apps which provide no symptom monitoring. Further research is needed examining different approaches for supporting people with chronic health conditions through ICBT, particularly looking at participant (e.g., different chronic health conditions, preferences) and intervention characteristics (e.g., with or without telephone assessment), that may influence engagement and outcomes.

## Data Availability

Not applicable.
